# Perspective on the human cough reflex

**DOI:** 10.1186/1745-9974-7-10

**Published:** 2011-11-10

**Authors:** Stuart M Brooks

**Affiliations:** 1 Colleges of Public Health and Medicine. University of South Florida, Tampa, Florida

**Keywords:** Human evolution, Cough mechanisms, Transient receptor potential vanilloid cation channel (TRPV1), Mechanosensory Cough, Chemosensory Cough, Urge to cough

## Abstract

This review dissects the complex human cough reflex and suggests hypotheses about the evolutionary basis for the reflex. A mechanosensory-induced cough reflex conveys through branches of myelinated Aδ nerve fibers is not chemically reactive (i.e., capsaicin, bradykinin); possibly, its evolution is to prevent the harmful effects of aspiration of gastric or particulate contents into the lungs. This became necessary as the larynx moves closer to the opening of the esophagus as human ancestors adapt phonation over olfaction beginning less than 10 million years ago. The second type of cough reflex, a chemosensory type, is carried by unmyelinated C fibers. Supposedly, its origin dates back when prehistoric humans began living in close proximity to each other and were at risk for infectious respiratory diseases or irritant-induced lung injury. The mechanism for the latter type of cough is analogous to induced pain after tissue injury; and, it is controlled by the identical transient receptor potential vanilloid cation channel (TRPV_1_). The airways do not normally manifest nociceptive pain from a stimulus but the only consistent response that capsaicin and lung inflammation provoke in healthy human airways is cough. TRPA_1_, another excitatory ion channel, has been referred to as the "irritant receptor" and its activation also induces cough. For both types of cough, the motor responses are identical and via coordinated, precisely-timed and sequential respiratory events orchestrated by complex neuromuscular networking of the diaphragm, chest and abdominal respiratory muscles, the glottis and parts of the brain.

## Background

Persistent cough is one of the most common medical complaints that impacts the quality of life and is responsible for a significant proportion of annual ambulatory medical visits and medical expenses in the United States [1]. Involvement of the upper and/or lower airways play pathogenetic roles in cough development and the association of allergy represent an important contributing factor for cough exacerbation. This perspective explores the complexity of coughing and suggests hypotheses about the unique evolutionary basis for the human cough reflex.

### Complexity of watching someone cough

Considering a casual connotation, coughing is a reflex-evoked modification of normal breathing patterns [2]. More explicitly, the cough reflex is a multifaceted, precisely timed, neuromuscular phenomenon characterized by the precise concurrent and sequential coordination of the activation patterns of the diaphragm, various muscle groups of the chest wall, cervical muscles, abdominal muscles, laryngeal abductor and adductor muscles, medullary and higher cortical regions of the brain [3,4]. The complexity of coughing is espoused by television personality Jerry Seinfeld when he informs his friend George Costanza (played by Jason Alexander): "*When you cough there are thousands of unseen muscles that suddenly spring into action. It's like watching that fat guy catch a cannonball in his stomach in slow motion*" (from *The Apology*, the 165th episode of the NBC sitcom *Seinfeld *that was first aired on December 11, 1997).

Accordingly, I am attending a dinner with my wife and observe her walking across the room towards me. As she does so, she aspirates a sliver of ice from a glass of beverage she is drinking. My wife stops walking and her eye brows arch. She informs me later that at that instance she experienced the sensation of an urge to cough. She deftly places a clenched fist to her mouth as her face reddens; her eyes water, narrow and then tighten. Her chin lifts to some extent as her head rears back; perspiration appears on her upper lip. Her stance widens. Her back bends slightly backwards. Her chest expands as she takes in a breath. She holds her breath momentarily only to quickly open her mouth again. Now, her face is cerulean-colored as she forcibly emits a staccato-like exhalation. Then, at the very end of her explosive exhalation, she daintily wipes a small speckle of ice from her lower lip using a pink-colored lace handkerchief that she inconspicuously hides in the cleavage of her dress. As she looks across the room at me, she smiles and nonchalantly lifts both her shoulders and spreads her arms out with the palms facing upwards as if to denote a sense of embarrassment.

### Sensory phase of coughing

#### Mechanosensory Cough

For my wife, it is reasonable to believe that instigation of a cough response is due to stimulation of a mechanically-sensitive 'true cough receptor' that is provoked by the sliver of ice. The premise for the presence of a 'true cough receptor' is explored by Canning et al using the anesthetized guinea pig model, which retains a blunted cough response following noxious stimuli [5,6]. Mechanical stimulation, postnasal drip, and a water bolus placed into the pharynx will evoke coughing in both human subjects and in animals but not capsaicin [6,7]. While C-fiber activation initiates coughing in conscious guinea pigs, it does not occur in the anesthetized animal. Anesthesia also attenuates or abolishes coughing in humans [8]. The anesthetized guinea pig model is important because it allows different investigative options, such as examining cough along with recordings from brainstem neurons or vagal afferent neurons, microinjection of drugs into specific brainstem and midbrain structures, selective stimulation of parts of the airways and not others, or extrinsic denervation of only parts of the airways [6]. My wife's coughing commenced with an inspiratory maneuver. In other cases, the mechanosensory-type reflex cough is associated with just a single and short expiratory cough referred to as an 'expiration reflex' [9-11].

Purportedly, the vagal afferent neurons of the true cough receptor are unlike the rapidly adapting receptors (RARs), slowly adapting receptors (SARs) or C-fibers; also, this receptor does not express transient receptor potential vanilloid (TRPV1) and is not sensitive to capsaicin. In contrast to the anesthetized guinea pig model, mechanically-induced coughing in conscious guinea pigs generates impulses carried by low threshold, mechanically sensitive, rapidly adapting receptors (RARs) that travel through myelinated Aδ fibers at conduction velocities of between 4 and 18 m/s [12-17]. These receptors also do not react directly to capsaicin and other chemical stimuli unless the stimulus leads to mechanical distortion of the nerve terminal [18]. RARs promote reflex bronchospasm and mucus secretion through parasympathetic pathways. How the conscious guinea pig cough mechanism applies to my wife's induced-coughing is not sufficiently understood.

Tracheal and laryngeal receptors come in to play as an important defensive role against acid aspiration [19]. The 'true cough receptor' is provoked by acid [6]. The descent of the larynx to a location more approximate to the opening of the esophagus places human ancestors at a greater risk for aspiration. Because of the greater risk for acid aspiration, possibly there is adaptation of a means to provoke coughing via a brainstem sensitizing mechanisms or by direct triggering of afferent esophageal nociceptors projecting from vagal pharyngeal and glossopharyngeal nerves [14,20]. The ion channel receptor, TRPV1, respond to acid in a more sustained manner than the acid-sensing ion channel-type receptor (ASIC), which tends to produce brief and transient responses to a lowered pH [21].

#### Chemosensory Cough

Both the chemically- and mechanically-sensitive airway nerves take part in mediating the cough reflex and establishing synapses in the brainstem's caudal two-thirds of the nucleus tractus solitarius [22]. Because the threshold for mechanical activation is more conducive for RARs and SARs than for C-fibers type of nerves, C-fibers consequently respond less to mechanical stimuli than do RARs and SARs. In some animals (i.e., guinea pigs, rabbits, cats, dogs and pigs) glutamate may be the final central nervous system excitatory transmitter during coughing; neurokinins play a more modulatory role [23]. The chemosensory nociceptors reside as a fine plexus within the airway epithelium and walls and send nerve impulses through slowly conducting (< 2 m/s) vagal unmyelinated fibers [24-28]. The sensation of an "urge to cough" is ostensibly associated with activation of bronchopulmonary C-fibers [29,30]. C-fiber nerves become directly activated, 'sensitized' or 'hyper-activated' by capsaicin, bradykinin, adenosine, PGE_2_, citric acid, hypertonic saline solution, SO_2 _and lung inflammation (or inflammatory-type chemicals) [7,18,31-33]. The role of lung neuropeptides and neuroinflammation in humans is poorly understood [34-37].

C-fibers actions involve ion channels, ancient sensors of the environment. Hundreds of millions of years ago, ion channels first sense thermal and osmolality stimuli. Later, the ionic channel mechanisms are adapted for other 'environmental' sensations (e.g., hearing, vision and taste). TRPA_1_, possibly an ''irritant receptor'', is expressed by a subpopulation of unmyelinated afferent C fiber nociceptors and may be linked to TRPV_1 _to contribute to the transduction of the irritant stimuli [38-43]. Mazzone questions the role of TRPV_1 _and opines that it is improbable that any TRPV_1_-expressing cells in other tissues or organs are involved in the cough reflex [18].

#### Motor Phases of Coughing

The motor pattern of the reflex cough is regulated differently than the motor pattern for tidal breathing [44]. Neuroplastic transformations allow respiratory behaviors having dissimilar spatial and temporal dimensions to instigate a mixture of actions employing comparable respiratory motoneurons [37,45]. Animal investigations concerning the induction of *Fos*-like immunoreactive neurons in certain brain stem regions (i.e., the commissural subnucleus of the nucleus tractus solitarius, field of the reticular formation, and nucleus ambiguus) suggest that different reflex responses make use of overlapping neural circuitry [46]. The neural conduit for medullary control of laryngeal adductor muscles actions is contained within a broader neural pathways controlling cough and swallowing [46]. In animal models, there is activation of interneuron pathways located between the medullary nucleus tractus solitarius and the nucleus ambiguus during coughing [46,47]. Stimulation of the superior laryngeal nerve can evoke different laryngeal adductor muscle responses including coughing, swallowing, gagging, laryngeal spasms, bronchoconstriction, apnea, and retching [46,47]. The type of the evoked reflex response depends on the considerations of the stimulus used [46,47].

**Figure 1:**
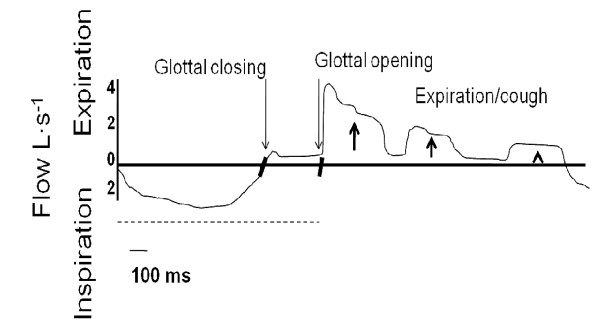
**Inspiratory and expiratory flow rates measured over the time course of a coughing event**. The figure displays inspiratory and expiratory flow rates measured over the time course of a coughing event. The inspiratory phase of coughing is when an inspiratory maneuver expands the lung volume for sufficient expiratory power. The second, compressive, phase entails narrowing or complete closure of the glottis that allows pressure to build up. During the brief closure, there is isovolume (i.e., no flow) contraction of the chest wall against a closed glottis. During the expulsive phase, there is glottal opening and the explosive release of a burst of air with expiratory cough rejoinders. There is the characteristic cough sound during the latter maneuver.

While conceivably not evident to my wife, the motor side of her cough reflex is spaced by distinctive phases (Figure 1). During the inspiratory phase of cough, my wife's entire glottis is in abduction. The laryngeal motor neurons that begin in my wife's nucleus ambiguus follow vagal and superior laryngeal nerves to excite the motor neurons of her glottis, external intercostal muscles, diaphragm, and other major inspiratory and expiratory respiratory muscles [48]. Her upper airway motoneurons are located at the cranial level; phrenic motoneurons controlling the diaphragm are located in the cervical cord ventral horn; and, her rib cage and abdominal motoneurons are located in the ventral horn of the thoracolumbar segments [49,50]. If measured, intracellular electrodes would record substantial depolarization of her laryngeal motor neurons during coughing. It seems that a central inspiratory command activates the respiratory motoneurons in an encoded sequential order; the upper airway motoneurons are recruited before those of the diaphragm and the rib cage; this allows the opening of the glottis before the fall in tracheal pressure due to diaphragmatic contraction [49]. The posterior cricoarytenoid muscle activates 40 to 100 milliseconds before the inspiratory activation of the diaphragm [51]. Actions taken by other inspiratory and the laryngeal abductor muscles (e.g., posterior cricoarytenoid muscle) further enlarge the opening of her upper airways [52-60]. My wife's diaphragm and external intercostal muscles contract to expand her chest cavity and lower her intra-thoracic pressure. The crural and costal muscles of her diaphragm act in synchrony throughout inspiration [61]. The contraction of her diaphragm peaks within approximately 1.0 second. As her diaphragm descends, there is some widening of her glottal opening due to tautness placed on her larynx [62].

The compressive phase of her cough reflex begins almost immediately after inspiration. Her laryngeal motor neurons are transiently hyperpolarized during the transition between the inspiratory and expiratory compressive phases of her cough; laryngeal motor neurons are repolarized during the expiratory compressive phase [9]. The glottic closure is essential for the process of coughing since the maximal level of intrathoracic pressure attained, and the efficiency of the expiratory cough, depends in great part on the quality of glottic occlusion. The glottic closure reflex is elicited at birth, becomes more active during the first year of life, and gradually decreases in activity with further aging [51]. Very quickly and in the order of perhaps 200 milliseconds and a range between 42 and 1010 milliseconds, the glottic closure takes place as two small laryngeal abductors (posterior cricoarytenoid muscles) quickly relax and her laryngeal adductors (e.g., thyroarytenoid muscles) contract to produce significant narrowing or complete closure of the glottis [3,63,64]. The laryngeal sphincter muscles closure is so tight that it can sustain very high intratracheal pressures [65]. Intrapleural pressure may reach more than 100 cm H_2_O [4,56,58,65,66]. There is coordinated activity between the posterior cricoarytenoid muscle and the accessory inspiratory muscles (i.e., infrahyoid and intercostal muscles) [51,57,67]. Synchronized actions are taken by her expiratory, abdominal and laryngeal adductor muscles. There is further activity by her diaphragm during the compressive phase of the cough reflex that physiologically translates as an isovolume (i.e., no flow) contraction of her chest wall against a closed glottis.

The expulsive phase of the cough reflex begins as the laryngeal adductor muscles (thyroarytenoid and arytenoideus) contract starting a few hundred milliseconds before the diaphragm relaxes and while the abdominal muscles have already relaxed [3,54,63]. An effective diaphragmatic force helps my wife to cough. There is inhibition of laryngeal adductor motoneurons, which is important in the generation of explosive expiratory airflow [64]. The posterior cricoarytenoid muscles briefly contract to enlarge the glottic opening but, not as wide as during the inspiratory phase; there is now a strong positive swing of pleural pressure. Her true vocal folds are pulled downwards during the explosive expulsive phase of coughing. The cricothyroid muscle causes vocal fold elongation and increased size of the glottic opening. A transiently relaxed glottis releases a burst of expired air to expel the piece of ice [64]. Finally, the larynx again constricts a bit and her diaphragm relaxes after coughing stops [54,64].

My wife's true and false cords are participants in coughing since the contraction of the thyroarytenoid muscles alters the position, shape and tension of her false cords. This allows their shelf-like, down-turned free margins to function as a one-way valve to prevent the escape of air from the lower respiratory tract below. In this way, it helps buildup intrathoracic pressure [56]. Her true cords, with their up-turned margins, behave as a one-way valve in the opposite direction, obstructing the entrance of air from above [56]. From a structural point of view, her false cords provide more of an expectorative function for cough, whereas her true cords assume a more protective role against aspiration [56].

My wife's distinctive cough sound is caused by oscillation of the surrounding lung/upper airways' tissues and gases related to the relatively large expiratory airflow velocities during the explosive exhalation [4,68]. The quality of the cough sound is influenced by airflow speed, changes in resonance of the airway tissues, secretions that are present in the airways and the compliance of the airways [63].

#### Brainstem Nervous Control of Cough

Multitudes of neural messages are integrated, interconnected and funneled to my wife's brainstem cough centers from peripheral afferent sensors that travel through the vagal internal laryngeal nerve to the medulla and interconnect with neural networks in the cortex. Vagal and glossopharyngeal motoneurons innervate her upper airway muscles; the inspiratory and expiratory bulbospinal pre-motoneurons, of the intermediate and caudal regions of the ventral respiratory group, project nerve impulses to phrenic, intercostal, and abdominal motoneurons [4,58,69]. Second-order neurons launch signals to her brain stem nervous systems that influence the normal respiratory cycle but also help carry out coughing [58,70]. The pontine and rostral ventral respiratory groups, the raphe nuclei and Bötzinger and pre-Bötzinger complexes adjust varied cough discharge patterns [58,70-72]. Apparently, the pre-Bötzinger complex helps generate inspiratory respiratory rhythm while the retrotrapezoid nucleus/parafacial respiratory group in front of it plays a role for implementing expiratory rhythms. Expiratory neurons, possessing augment firing patterns, span regions of the Bötzinger and pre-Bötzinger complexes to initiate and inhibit premotor neurons of the laryngeal adductor muscles [71,73-75]. Possibly, an endogenous cough-suppressing neuronal network located within her caudal ventral respiratory region plays some role in modulating the excitability of the cough reflex [37,69,76,77]; there could be some sort of gating mechanism operating at some level [78,79].

#### Cortical Nervous Control of Cough

In humans, the cortical participation of the cough reflex is not like any other animal [5]. Accordingly, my wife is capable of controlling her forced exhalation during coughing. She has the ability to voluntarily initiate or inhibit her cough responses without sensory stimulation, during capsaicin inhalation and also with upper respiratory tract infections [32,60,80-82]. My wife's cough is lost or severely diminished during general anesthesia or sleep; and, systemic opiates suppress her coughing [7,30,33,60,83]. Her cough is susceptible to placebo-induced suppression [29,30,32,84]. She notes an "urge-to-cough" that always precedes her actual cough motor maneuver [84]. If she is investigated by imaging studies during voluntary coughing, the findings likely will show brain activity appearing in the ventrolateral sensorimotor cortex, an area responsible for non-respiratory orofacial (i.e., chewing, lip pursing and tongue movements) and respiratory orofacial movements (i.e., speaking, singing, and swallowing) [81]. More recently, Mazzone and colleagues, measured blood oxygen level-dependent (BOLD) responses in human subjects utilizing the technique of event-related functional magnetic resonance imaging and confirmed that the largest areas of imaging during voluntary coughing occurred in cortical areas functionally linked to both respiratory-related orofacial tasks (i.e., speaking and singing) and also non-respiratory orofacial actions (i.e., chewing, lip pursing, and tongue movements) [33].

## Discussion

Coughing can be provoked in a variety of animal models, such as the guinea pig, cat, dog and pig [85]. Yet, there are distinctive differences between humans and these animals as well as non-human primates. The distinguishing differences between non-human and human cough reflexes are the results of transformations that take place over millions of years in part responsive to changes in the internal and external environments of evolving humans [5,20,86-93]. The scheme utilizing coughing dates back millions of years before when a very primitive mammal first employs this defense [6,20,94,95]. Alternatively, the repositioning of the larynx in the hominid linage may have been a gastrointestinal adaptation in the ape swallowing mechanism because of the separation of the gastrointestinal system from the respiratory tract [96]. The crural diaphragm may have been a gastrointestinal sphincter to defend against gastroesophageal reflux and aspiration [61,97].

**Figure 2:**
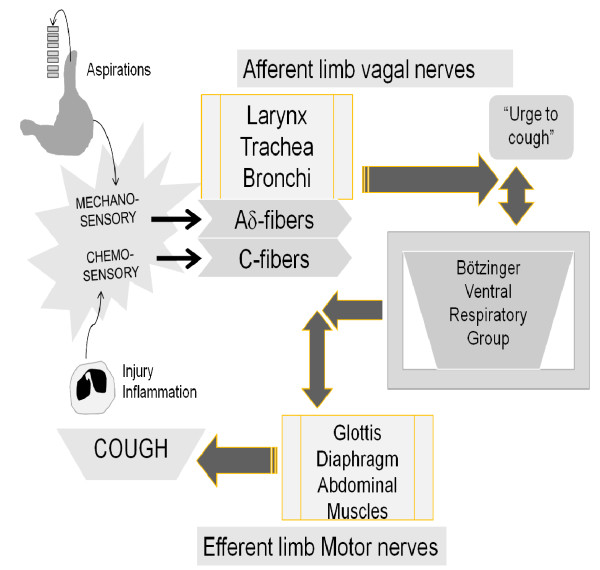
**Types of Cough Reflex**. The pattern of the cough reflex depends on the nature of the evoking stimulus as well as on the part of the respiratory tract stimulated. There are two or more distinctive human cough reflexes with likely different evolutionary histories. The first is the involuntary cough reflex provoked by mechanosensory-responsive Aδ fibers. It is relatively insensitive to chemical stimuli and was derived as a protective mechanism against aspirated gastric content due to the change in the position of the larynx in humans and their primate ancestors. This type of cough reflex evolved a few million to several hundred thousand years ago. An alternative possibility is what has been identified in both conscious and anesthetized guinea pigs and presumably also in humans called the "true cough receptor" as described in originating from low threshold mechanosensitive afferent nerve fibers and is activated by light punctuate mechanical stimulation and rapid changes in pH evoke cough [6,18]. The rapid changes in pH may hold for the protection against aspiration of gastric acid. The second type of involuntary cough reflex occurs when unmyelinated vagal sensory C-fibers are activated by tissue injury, inflammation or by chemicals such as capsaicin, bradykinin, SO2, and citric acid. The chemically-sensitive cough reflex was possibly a co-evolutionary response by different animal and plant species and occurred over hundreds of millions of years.

Feasibly over millions of years, the revisionary wings of evolution respond to changes in the hominids' environments as two different cough responses emerge (Figure 2). Over this period of time, genes are duplicated and/or reused, with minor modification, either in the same hominid or a more primitive mammal [98-101]. The regulation of the cough reflex differs greatly from that of tidal breathing [79]. Neuroplastic transformation refigures basic respiratory behaviors, in some very ancient mammal, in order to utilize the same muscles and nerves of normal breathing for the cough reflex [47]. The enlarging brain and changing supralaryngeal tract are important evolutionary drivers for human speech and higher cognitive functions that impacts on the human cough reflex [33,47,78,102-105]. A more archaic *Homo sapiens *with a larger brain and a changing supralaryngeal tract emerges about 500,000 years ago [106-108]. While the ancestor of the very earliest *Homo *species may have been chimp-like, it takes serial hominid intermediaries of at least 15-20 "chronospecies", spread out over 6-7 million years, before the modern-type human appears in Africa, between 200,000 and 100,000 years ago [98,109,110].

The foremost driving forces for one type of *Homo *species cough reflex are modifications of the supralaryngeal tract, decent of the hyoid bone and movement of the larynx closer to the esophageal opening. These changes necessitate a cough defense against aspiration. In an evolutionary sense, the adaption of a neural mechanism such as a 'true cough receptor' (or a similar mechanism) becomes a valuable pulmonary defense. Generating complex sounds utilizing the laryngeal sphincter as a vibratory source is a unique evolutionary adaptation of humans [51]. The distinctiveness of the human glottis is supported by electrophysiological measurements of laryngeal muscles movements during voice maneuvers. In humans, there are peak electrical activities in cortical *motor area 4*; non-human primates show electrical peaks in cortical *motor area 6 *[81,111]. Also, as *Homo *species evolve, the margins of the human vocal cords lose some of their sharpness; arytenoid cartilages became smaller and vocal folds elongate to produce a wide range of sounds. For my wife's pre-*Homo *species ancestors, living millions of generations before, the additions of small arytenoid cartilages to the tips of the true vocal cords increases its relative length and maximize its vibratory surfaces [56]. An arytenoid length of 7 compared to a true cord length of 10 (7/10) represents the best cross-sectional area of the glottis; this is when there is ultimate pivotal movement of the arytenoid bodies [56]. The 7/10 ratio allows for the widest laryngeal opening and airways possessing the lowest airflow resistance possible. Such an optimum ratio of arytenoid to vocal cord length is found only in racing animal that need to be able to run fast, such as the gazelle. In contrast, humans who do not depend on flight for protection possess a less efficient 4:10 ratio [56].

**Figure 3:**
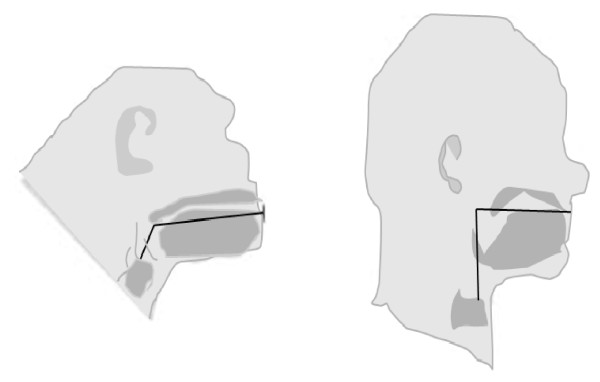
**The *Homo *species supralaryngeal tract**. The *Homo *species supralaryngeal tract (right) is characterize by horizontal portion (i.e., mouth and oropharynx) forming a right angle of approximately equal lengths of 1:1 proportion with a vertical segment extending down from the palate to the vocal cords. In comparison, the chimpanzee (left) has a hyoid bone and larynx positioned at or near the base of the mandible; and, the tongue is long and mostly restricted to the oral cavity, resulting in a disproportionate supralaryngeal vocal tract. Human ancestors such as *Homo erectus *and Neanderthal chronospecies possessed supralaryngeal vocal tracts intermediate in shape between those of chimpanzees and humans. (Adapted from [108]).

For the evolving hominid, the sounds exiting from the mouth are tailored by the "supralaryngeal vocal tract" shaped by two portions that form a right angle to one another (Figure 3) [108]. The earliest hominids likely possesses a supralaryngeal vocal tract having its horizontal dimension longer than its vertical one, making them incapable of producing the full range of sounds made by humans today. Only for the *Homo *species is there a unique descent of the hyoid bone to well below the mandible [108]. In comparison, the chimpanzee's hyoid bone and larynx position at or near the base of the mandible; and, the chimpanzee's tongue is long and mainly limited to the oral cavity, resulting in a disproportionately shaped supralaryngeal vocal tract. Human ancestors such as *Homo erectus *and Neanderthal chronospecies possess supralaryngeal vocal tracts intermediate in shape between those of chimpanzees and humans. Over time, the changing positions of the tongue, lips, and larynx alter the overall configuration of the supralaryngeal vocal tract; such transformations place humans at a greater risk for aspiration [96,108]. The exact impact on the cough reflex of the evolutionary shaping of the supralaryngeal tract and humans' faculties for better cortical control of the muscle movements of the larynx, tongue, mouth and lips have not been adequately explored.

The growth in the size of the human cortex permits better control of voluntarily speech and song production (and laryngeal muscle movements) as opposed to the limited voluntary control over vocalizations displayed by non-human primates [102,103,112-114]. Hundreds of millions of years before, the larger brain size of ancestral mammals compared to their closest extinct mammalian relative is in response to the high resolution of olfaction, prominence of odorant genes and growth of odorant receptors [104]. The relationship between keen olfaction and brain size is changed in the *Homo *species as they adapt speech over olfaction. The genes regulating brain size and behavior exhibit higher rates of protein evolution in the lineage leading from ancestral primates to humans [112]. The abnormal spindle-like microcephaly-associated gene (*ASPM*) may have been the evolutionary target for the initial expansion of the hominid cerebral cortex; and, changes regarding human speech are likely accelerated after the FOXP2 regulatory gene reaches its modern normal variant around 100,000 years ago [112,115-123].

The second type of human cough reflex is adapted as the capacities for speech and cognition evolve and *Homo *species fashion stronger social connections. There emerges an imperative need to defend against distal lung inflammation or damage as *Homo *chronospecies move to more enclosed environs where there is a greater possibility of contracting a contagious respiratory tract or parasitic infections and/or being exposed to gaseous-particulate irritant emanations [124,125]. Pulmonary inflammation often accompanies a contagious respiratory tract infection or following an irritant inhalational exposure [3,7,20,124-127]. The earliest community sites for the ancient *Homo *species, possibly beginning 400,000 years, are supposedly within caves requiring fire for warmth and for cooking of food [128]. Fire requires the burning of fuels in the forms of biomass, such as wood, animal dung and crop residues [129]. Biomass smoke composed of irritant particles and gases can penetrate deeply into the lung to produce a variety of inflammatory morphologic and biochemical changes.

Teleologically, the lung C-fiber neural responses may permit a broader defensive rejoinder than does just coughing, a warning sign like pain. Possibly, cough associated with respiratory infections is a coevolutionary strategy by primitive viruses coexisting with human predecessors; induction of coughing increases viral contagiousness to enhance viral spread and survival [124,130]. The coughing part is driven by the virus while neuroinflammation or some other process (in humans) represents a type of innate immunity. Maybe, neuroinflammation corresponds to a first-line inflammatory defense until the actual immune inflammatory response against the respiratory tract infection begins [131,132]. Unfortunately, while documented in guinea pigs, lung neuroinflammation in humans is not well delineated [127,133-138].

## Summary

A mechanosensory reflex cough, receptive to mechanical and acid stimulations, possibly operates via a mechanism involving the so-called "true cough receptor"; or possibly, there is another mechanism employing other slowly conducting nerve fibers. Pertinent evolutionary adaptations shaping the first type of human reflex cough response comprise modifications of the supralaryngeal tract and descent of the hyoid bone and movement of the larynx to a closer location to the esophageal opening, which increases the risk for aspiration. A second, chemosensory-type reflex cough, originating in some more ancient mammal, involves C-fiber afferent nerves linked to lung inflammation and chemical agents (i.e., capsaicin, bradykinin, etc.). The need for defending against distal lung inflammation or damage emerges as *Homo *chronospecies move to more enclosed environs where there is a greater possibility of contracting a contagious respiratory tract or parasitic infections and/or exposure to gaseous-particulate irritant emanations. *Homo *species fashion stronger social connections with the introduction of speech (over olfaction) and the enlargement of the cortical brain size. *Homo *species (and perhaps an earlier hominid) achieve voluntarily cough initiation and suppression; control of the forced exhalation and the intensity of the cough response; the facility to initiate repeated coughing; a heeding to the integration of psychosocial factors into the cough response; exquisite control of laryngeal muscles actions; a capacity to respond to a variety of cough stimuli; and the perception of airways irritation causing an "urge to cough".

## List of abbreviations used

**TRPV_1_**: Transient Receptor Potential Cation Channel Subfamily V, Member 1; **TRPA_1_**: Transient Receptor Potential Cation Channel, Subfamily A, Member 1; **Aδ Nerve Fibers: **A Delta Fibers (Afferent Fibers); **C Nerve Fibers: **Unmyelinated C fibers (Afferent Fibers); **SARs: **Slowly adapting Fibers; **RARs: **Rapidly Adapting Receptors; **ASIC:**Acid-Sensing Ion Channel-Type Receptor; **PGE_2_**: Prostaglandin E2; **BOLD:**Blood Oxygen Level-Dependent; **FOXP_2_**: Forkhead Box Protein P_2_

## Competing interests

The author declares that they have no competing interests.

## Authors' information

Dr. Stuart M Brooks is currently Adjunct Professor in the College of Public Health, University of South Florida.
